# A Scoping Review of Digital Tools to Reduce Sedentary Behavior or Increase Physical Activity in Knowledge Workers

**DOI:** 10.3390/ijerph17020499

**Published:** 2020-01-13

**Authors:** Ida Damen, Hans Brombacher, Carine Lallemand, Rens Brankaert, Aarnout Brombacher, Pieter van Wesemael, Steven Vos

**Affiliations:** 1 Department of Industrial Design, Eindhoven University of Technology, 5600 MB Eindhoven, The Netherlands; j.g.brombacher@student.tue.nl (H.B.); c.e.lallemand@tue.nl (C.L.); r.g.a.brankaert@tue.nl (R.B.); a.c.brombacher@tue.nl (A.B.); 2 HCI Research Group, University of Luxembourg, 4365 Esch-sur-Alzette, Luxembourg; 3 School for Allied Health Professions, Fontys University of Applied Sciences, 5600 AH Eindhoven, The Netherlands; 4 Department of the Built Environment, Eindhoven University of Technology, 5600 MB Eindhoven, The Netherlands; p.j.v.v.wesemael@tue.nl; 5 School of Sport Studies, Fontys University of Applied Sciences, 5644 HZ Eindhoven, The Netherlands

**Keywords:** technological interventions, workplace, knowledge workers, scoping review, physical activity, sedentary behavior

## Abstract

*Background:* There is increasing interest in the role that technology can play in improving the vitality of knowledge workers. A promising and widely adopted strategy to attain this goal is to reduce sedentary behavior (SB) and increase physical activity (PA). In this paper, we review the state-of-the-art SB and PA interventions using technology in the office environment. By scoping the existing landscape, we identified current gaps and underexplored possibilities. We discuss opportunities for future development and research on SB and PA interventions using technology. *Methods:* A systematic search was conducted in the Association for Computing Machinery digital library, the interdisciplinary library Scopus, and the Institute of Electrical and Electronics Engineers Xplore Digital Library to locate peer-reviewed scientific articles detailing SB and PA technology interventions in office environments between 2009 and 2019. *Results:* The initial search identified 1130 articles, of which 45 studies were included in the analysis. Our scoping review focused on the technologies supporting the interventions, which were coded using a grounded approach. *Conclusion:* Our findings showed that current SB and PA interventions using technology provide limited possibilities for physically active ways of working as opposed to the common strategy of prompting breaks. Interventions are also often offered as additional systems or services, rather than integrated into existing office infrastructures. With this work, we have mapped different types of interventions and provide an increased understanding of the opportunities for future multidisciplinary development and research of technologies to address sedentary behavior and physical activity in the office context.

## 1. Introduction

A crucial factor that negatively affects a person’s vitality is the lack of physical activity (PA) and high levels of sedentary behavior (SB) throughout the day [1]. Prolonged periods of sedentary behavior, characterized by waking behavior equal to or below 1.5 METs [2], can have a severe negative effect on health [3,4,5]. Sedentary behavior is associated with the development of diseases such as type II diabetes, cardiovascular diseases, colon and breast cancer, increased morbidity, and premature mortality [3,5,6,7,8,9,10]. Our increasingly sedentary lives have thus become a major public health risk [11]. Conversely, engaging in physical activity, defined as any body movement that raises energy expenditure above resting metabolic rate [12], is positively linked to improved vitality [1].

To combat the negative health effects of prolonged sitting, it is important to integrate physical activity into daily routines and reduce the amount of sedentary behavior. This, however, proves difficult in office contexts. We currently spend up to 71% of our working hours sitting [13], and trend analysis indicates that sedentary behavior will continue to increase in the near future [14]. To counteract this trend, there is a growing need to investigate how to increase PA and reduce SB in office settings. In addition, a growing body of work shows that increasing and embedding physical activities in work routines does not only increase workers’ health and wellbeing, but also improves social interaction and work performance [15]. 

A myriad of interventions targeting sedentary behavior or physical activity in workplace contexts have been developed by researchers from different disciplines. In recent years, several systematic reviews have assessed these interventions [16,17,18,19]. The reviews have shown that only a small portion of the interventions that were assessed had a digital component. This is despite the fact that digital technologies have the potential to “revolutionize the way individuals can monitor and improve their health” [20]. For instance, one might use a personalized services such as Google Goals, a calendar application to integrate personal goals into a user’s digital calendar [21]. These types of tools may increase reflection on personal goals and thereby advance the effectiveness of the intervention [22]. 

Few reviews could be found that focused on or included interventions using technology. In a systematic review on the effectiveness of SB workplace interventions by Chau et al. [23], three studies had a technological element [23,24,25,26] consisting of either email or the use of a pedometer. Another four studies were included in a review by Shrestha et al. [27], involving computer prompts [28,29,30] and e-newsletters [31]. In a study by Bort-Roig et al. [32] on smartphone strategies to influence physical activity, a total of 17 articles were reviewed. This study concluded that smartphone strategies tended to be ad hoc rather than theory-based [32]. 

In 2017, Stephenson et al. [15] systematically reviewed computer, mobile, and wearable technology SB interventions for healthy adults and found that these technologies can be effective in reducing SB. Their meta-analysis of 15 studies showed a mean reduction of 41 min/day in the intervention group at end-point follow-up. Another systematic review focusing solely on digital SB interventions was conducted by Huang et al. in 2019 [17]. This work differed from previous reviews by classifying technological features and annotated technological configurations of the interventions. In addition, they included engineering and computer science literature, while other reviews mainly included health and life science literature. 

Including interventions from the field of engineering and computer science, but also the fields of industrial, architectural, and urban design research is of great importance when developing an understanding of the opportunities for future development of SB and PA interventions. It is in these fields especially that we see the development of new and explorative approaches and interventions using technology. By rapid prototyping, development, and piloting of novel digital technologies, these fields may lay the groundwork for the development of future health-promotion technologies. 

In this paper, we reviewed recent interventions with a technological component aimed at reducing sedentary behavior or increasing physical activity of office workers. We included interventions from the fields of human computer interaction, engineering, computer sciences, and digital health. In our search, we not only included full text articles, but also case studies and work-in-progress papers. With this approach, we aimed to incorporate the latest ideas and developments in PA and SB interventions with a focus on the design of the interventions. Rather than focusing on the outcomes of the interventions, this review aimed to provide an overview of trends in how SB and PA interventions are shaped. By analyzing current approaches and trends in the form and function of the interventions, we identified underexplored possibilities and gaps for the design and development of future interventions and tools. We furthermore provide an increased understanding of the opportunities for future development of research and technologies that address sedentary behavior and physical activity in the office context.

The findings of this review can inform both the public health and design research fields, and address how the different fields can benefit from each other’s work. An increased understanding between these research fields and multi-disciplinary expertise is crucial in the development and evaluation of digital health interventions [33]. This review therefore provides considerations for the development of future SB and PA interventions using technology, as well as implications for more collaborative and interdisciplinary work utilizing these interventions.

## 2. Materials and Methods

### 2.1. Search and Selection

A literature search was conducted in the Association for Computing Machinery (ACM) digital library, the interdisciplinary library Scopus, and the Institute of Electrical and Electronics Engineers (IEEE) Xplore Digital Library. The search included full-text scientific articles, case studies, and late-breaking work published between January 2009 and June 2019. All libraries were searched for designs or interventions aimed at increasing PA or reducing SB of knowledge workers using digital technology.

A search strategy was performed on title, abstract, and keywords using the following keywords: “sedentary” OR “sitting” OR "physical activity" OR “inactivity” AND “office OR work*” AND “intervention” OR “design” OR “present*” OR “propose”. The search excluded “children” and “patient*”. To adjust the search to the nature of the different databases, two additions were made to the search queries. To limit the search in Scopus to designs with a digital component, the string “technology” OR “digital” was added. For the IEEE search, an extra exclusion string was added to exclude all SAT- and car-related papers. In addition to the database search, reference lists of existing reviews [15,17,24] on workplace SB reduction and PA promotion were manually searched to identify additional eligible studies. Titles and abstracts were reviewed for eligibility based on the following including criteria:

1. Presents a design targeting reduction of SB or increasing PA or both;
2. Partly or exclusively during office hours;
3. Including digital technology in the delivery;
4. Published in peer-reviewed scientific journals or conference proceedings between 2009 and June 2019;
5. Published in the English language.

The selection of studies was done independently by the first two authors based on a screening of titles and abstracts. The selected papers were cross-checked, and any discrepancies were resolved by including a third author to reach a consensus about study inclusion.

### 2.2. Data Synthesis

A grounded approach was used to develop a coding scheme for analysis of the designs. Two authors independently coded nine designs for an initial coding scheme. Consensus on the scheme was sought by including a third and fourth author. Based on the coding scheme, all eligible studies were reviewed and the designs were annotated. The following information was extracted: publication data, design details like mode of delivery, underpinning theories, behavior change techniques, objectives, targeted behavior, and details on the input and output of the design. 

## 3. Results

### 3.1. Study Selection

**Figure 1:**
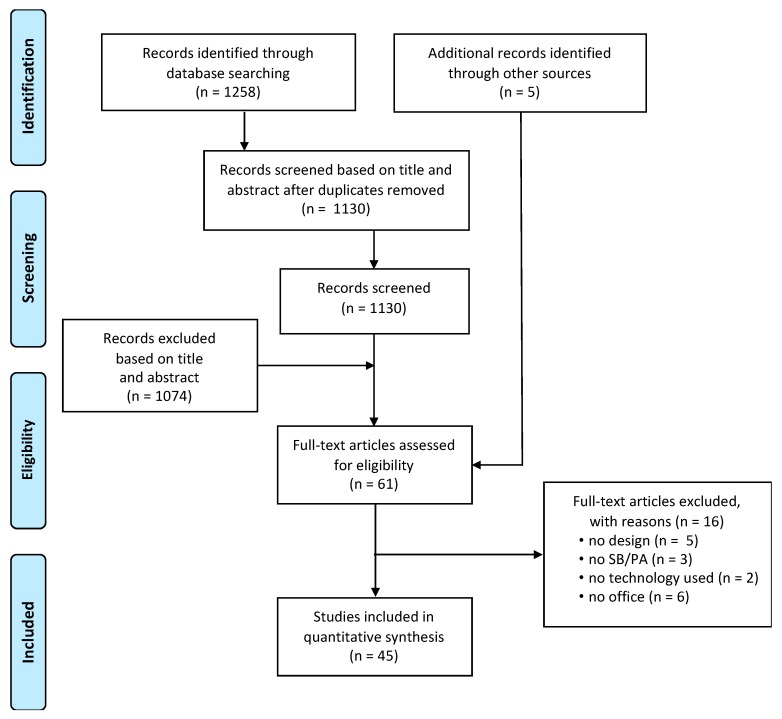
Selection of studies: PRISMA (Preferred Reporting Items for Systematic Reviews and Meta-Analyses) flowchart.

A structured database search identified 1258 potentially relevant abstracts (Figure 1). After removing 128 duplicates, 1130 unique references remained. The titles and abstracts of these articles were screened, after which 61 articles were selected for potential inclusion (Figure 1). Out of those, papers were excluded if there was no design presented (n = 5), if it had no digital component (n = 2), if the intervention did not target SB or PA (n = 3), or if it did not partially or fully target the office environment (n = 6). In total, 45 relevant articles were included in this review (Table 1).

### 3.2. Submission Type and Venue

**Figure 2:**
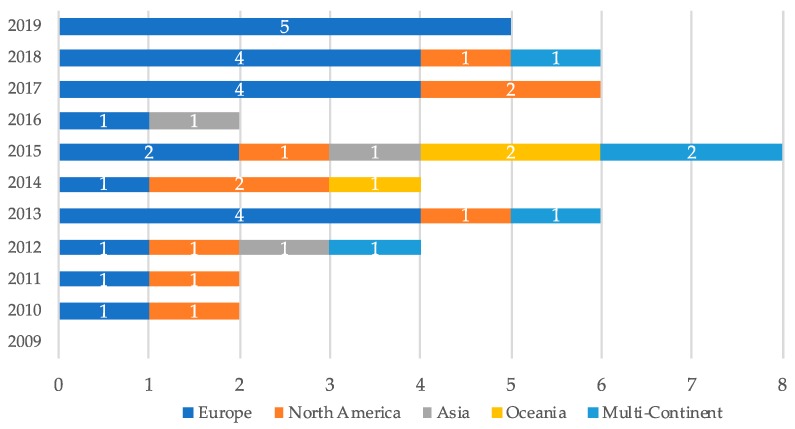
Number of included articles by year of publication and continent of authors.

Of the 45 included papers (Figure 1), 9 were full text journal articles, 22 were full-text conference proceedings articles, and 14 were short (work-in-progress) conference papers. The 45 included papers presented 47 unique designs to combat physical inactivity. The studies were published in 27 different journals or conference proceedings between 2010 and 2019 (Figure 2) by 41 different first authors. Twenty-four studies were published in the field of human–computer interaction (HCI), 12 studies originated from computer science journals or conference proceedings, and 9 studies were published in the field of digital health. However, it must be noted that many of the journals and conferences had an interdisciplinary character; it was thus complex to make a clear demarcation of the research fields to precisely categorize the papers in this review.

### 3.3. Theoretical Underpinning and Behavior Change Techniques

Of the 45 studies, 34 did not specify a theoretical underpinning of their design. Of the 11 papers that did use a theoretical model, the most commonly used theory was the transtheoretical model (25%) [37,58,68], followed by the theory of planned behavior (17%) [41,46] and self-determination theory (17%) [50,74]. Five other papers based their designs on, respectively, persuasive system design model [65], goal setting theory [55], distributed prospective memory approach [48], social cognitive theory [38] and social learning theory combined with the theory of reasoned action [46]. 

Eighteen different behavior change techniques (BCT) were specified in the papers, although 17 studies did not specify any BCT (Table 2) The most commonly used BCT was the use of rewards, for instance through gamification processes (e.g., earning points). Another common strategy, included in nine papers, was to include social support or sharing experiences with peers in the intervention.

### 3.4. Targeted Behavior

Twenty-six interventions were specifically designed for the office environment, while 19 studies had a more generic approach in which they did not specify the context of use. Eight papers reported an evaluation of their intervention on SB or PA [35,37,43,65,66,77,78]. Thirty-three studies did not give a definition of PA or SB, while nine studies provided a definition of PA and four studies offered a definition of SB (Table 3). 

Although the majority of the papers did not provide a definition of SB or PA, a general distribution of the main targeted behavior could be made based on the description of the interventions. Just over half of the interventions targeted PA (24) while 9 interventions targeted SB and 12 targeted both PA and SB. More specifically, 18 interventions targeted break-taking behavior, 20 interventions targeted non-work-related PA, 3 interventions targeted work-related tasks, and 4 interventions were categorized as “other targeted behavior”. The studies that were categorized as other targeted behavior included an intervention to moderate and maintain cadence [58], one intervention that stimulated connectedness using an ambient light system and PA visualizations on a screen [49], and two designs that targeted multiple lifestyle factors including diet, sleeping habits, and PA [37,46]. Of the three work-related behavior interventions, two designs aimed to stimulate walking meetings [41,49] and one intervention used movement on an interactive chair to control a workplace computer [69]. 

Another distinction could be made in the different approaches the interventions adopted to obtain the targeted behavior, namely creating awareness, creating opportunities, and teaching new behaviors (Table 4). The number of interventions that were classified as “creating awareness” exceeded the number of papers that stated intent to use awareness as a BCT, for instance because they used prompts to inform the user of prolonged sitting behavior. These interventions were categorized as creating awareness, even though this was not stated in the paper as a BCT. Twenty-three interventions aimed to create awareness of the user’s current behavior to let them reflect and act on their behavior. The most commonly used form of delivery was via prompting or messages. This strategy was adopted by 18 interventions. Seven interventions aimed to create opportunities for users to perform the targeted behavior [34,38,41,52,58,69,76]. Only one intervention aimed to transform old behavior into new, healthier behavior. This study by Probst et al. [69] provided office workers with the possibility to use tilting, rotating, or bouncing movements on an interactive chair to control their workplace computer. 

### 3.5. Technology Type

**Figure 3:**
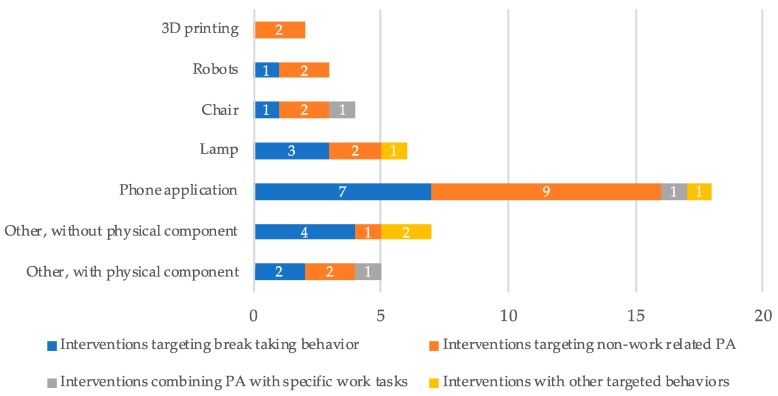
Approaches in targeted behavior in the included interventions with a physical component.

Several types of technology were used in the interventions (Table 5). Nineteen studies presented an intervention with a physical component (42%), such as a robot or an interactive lamp, while 26 of the studies (57.8%) showed purely digital designs like applications or computer software. Of the 19 studies with a physical component, 6 used a lamp as their primary feedback mechanism, 3 used a chair, 3 designs used robots, and 2 used 3D printing to represent physical activity (Figure 3; examples in Appendix A). Of the five remaining interventions with a physical component, one intervention used an ambient desk object [61], one intervention used an interactive fountain [57], one intervention integrated a physical line in a service design for walking meetings [41], and one was an interactive shirt [51]. 

Twenty-four interventions were purely digital, of which phone applications were most common. Phone applications in combination with wearables were often used to give information or prompts, and as a data collection tool. Seven interventions did not have a physical component, of which one used break-prompting software [60], one used a projected avatar [76], one was an email-based intervention [35], and four were platform-based interventions [37,44,46,47]. Figure 3 shows the different types of technologies used by the interventions in relation to the targeted behavior. 

Several types of objective data were collected as input measures by the interventions. Twenty-one interventions used step count as input, six measured heartrate, eight used motions or gestures as input, and three calculated caloric intake or energy expenditure (Table 6). Thirteen interventions used real-time data collection in their interventions. 

Of the 45 interventions, 7 employed existing infrastructures or tools. The tool most commonly used to build upon was the office chair [12,69,71,72]. Two interventions used office communication software in their design, by means of email [35] or a room booking system [41]. One intervention used a social communication channel (i.e., Facebook) as a platform for their design intervention [44].

In addition to the seven studies that used existing tools and digital services, there were two studies that restructured the built environment. The intervention “Breaksense” by Cambo et al. [40] introduced a Bluetooth beacon infrastructure to promote context-aware physical breaks within the workplace. Bluetooth beacons are low-energy devices that enable portable devices such as phones to be identified. By using this structure, Breaksense was able to trigger an action if a knowledge worker was close to a beacon, thereby encouraging workers to explore the office environment. Damen et al. [43] restructured the office campus environment by adding a physical line to their service design for walking meetings. 

## 4. Discussion

This review set out to scope the landscape of technological sedentary behavior (SB) and physical activity (PA) interventions used in the office environment. Through a systematic literature search in the Association for Computing Machinery digital library, the interdisciplinary library Scopus, and the Institute of Electrical and Electronics Engineers Xplore Digital Library, we identified and analyzed 45 interventions published between 2009 and 2019. This paper provides an overview of the study characteristics, including a description of the interventions, intervention objectives, theoretical underpinning, and behavior change techniques. The analysis revealed two important gaps in the current research and development of technological SB and PA interventions. 

A first underexplored opportunity in current interventions is the use of existing infrastructures. Seven studies employed existing office tools or systems, like chairs and emailing software. More often, interventions are offered as additional systems, tools, and services—an extra application on a phone or a robot is used to deliver SB or PA interventions. In future investigations, it might be possible to examine whether integrating interventions into existing office infrastructure will lower the threshold for people to use a system. Using existing systems and tools might limit additional the time investment required of users, which is an important design consideration for health-promotion interventions [11].

Second, the results showed that a clear distinction could be made in the objectives of the interventions. While a frequently adopted strategy of the interventions was supporting break-taking behavior, only a handful of studies attempted to create new, more active ways of working, i.e., how work can be transformed to be more physically active by making physical activity a more integral part of work and not merely a break from work, for instance by targeting walking meetings [41,79]. Only three studies targeted work-related tasks by transforming sedentary work behavior into physically active work behavior [41,69,79]. This finding was consistent despite the increased interest in sustainable performance at work, which extends the focus of worker health and wellbeing to health and wellbeing while maximizing work performance [82]. Interventions that focus on integrating physical activity with work may be more suitable to maximize work performance, compared to interventions that approach physical activity as a break from work. However, more work is needed on how SB and PA interventions can link to work performance. 

In addition, the two most common intervention strategies in our review were rewarding “good” behavior and creating awareness of “bad” behavior. Other frequently used techniques included goal setting, persuasion, and creating social support. The use of these behavior change techniques is in line with previous work on effective strategies in SB interventions by Gardner et al. [83] and Bort et al. [32]. These reviews found that persuasion [83], goal setting, and social support [32] were among the most promising behavior change techniques for SB interventions. Other highlighted strategies were environmental restructuring (i.e., changing the physical or social context), education and training, self-monitoring, and problem solving [32,83]. However, these techniques were only seldom used in the interventions included in this review. Moreover, eight of the included studies did not specify any behavior change techniques. In future work, researchers should thus strive to formulate behavior change techniques more clearly to create a more unified terminology for intervention strategies. 

In addition to formulating behavior change techniques, more clarity is needed on how SB and PA are defined within interventions. This review showed that a mere 12 of the included 45 interventions provided a definition of SB and PA. By adopting a unified terminology, a more profound exchange can be made between studies detailing development of interventions and evaluation studies. This can facilitate the adoption of novel technologies and interventions from design and engineering fields to be used in other fields. This is particularly important since the research and development of technological health interventions requires a multidisciplinary approach [33]. 

Our scoping review had a couple of limitations. First, by restricting our search to three databases, we may have excluded relevant publications. Similarly, by limiting our scope to scientific literature, we may have missed interventions that are used in practice but have not been reported on in the academic literature. We did, however, provide a broader scope than previous work by including short papers. By including exploratory or “late-breaking” work, we increased the chances of a more diverse scope of novel technologies being reviewed in this study. Focusing our review mainly on artefacts rather than studies, we obtained an overview of the strategies used and underused to address SB/PA in the context of office work. Although including short papers may have affected the level of information on interventions—and thus could have resulted in a lower overall quality of the included studies—this did not impede us in attaining the objectives of our study. Positioning this work as a scoping review of artefacts, we did not intend to report on the evaluation and impact of these technological interventions, and thus did not report on the methodological quality of the included studies or their reported results. Our present contribution instead focused on technology characteristics and identified current gaps and opportunities for future research and development of SB and PA interventions using technology.

## 5. Conclusions

In this investigation, we aimed to review the state-of-the-art technology interventions to reduce sedentary behavior or increase physical activity in the office environment, published in the fields of human computer interaction, engineering, computer science, and digital health and life sciences between 2009 and 2019. We included 45 interventions, identified using a systematic literature search. This study identified two main underexplored gaps and opportunities for future research and development of SB and PA interventions. With our work, we showed that current interventions make limited use of existing infrastructures and systems. The second major finding was that physical activity is approached as a break from work instead of an alternative, more active, way of working. Future work could investigate how physical activity can used as an active way of working instead of as a break from work. This new understanding should help to improve the understanding of current practice and provide opportunities for future research and development of SB and PA interventions. In addition, this work aimed to enhance the exchange of knowledge between different research fields and research phases by including papers reporting on the development of interventions as well as evaluation studies. We hope to improve a mutual understanding of the current SB and PA intervention practice, and thereby improve multidisciplinary work within the field of digital SB and PA interventions. Moreover, the list of artefacts can serve as an inspiration source for future development, and may help designers and developers to locate possibilities for novel design ideas [80].

## Figures and Tables

**Table 1:** Descriptions of included studies.

	Study	Name of the Design	User Evaluation (n)	Description Design	Objective(s) of the Intervention
1	Ahtinen et al. [34]	Brainwolk	11	Walking meeting concept mediated with a mobile app	Encouraging and supporting the practice of walking meetings
2	Andersen et al. [35]	N.S.	160	Email-based encouragements	Encouraging daily stair-walks together with colleagues
3	Arrogi et al. [36]	stAPP	56	Smartphone application	Interrupting and reducing prolonged sitting behavior
4	Bonn et al. [37]	Health Integrator.	209	Platform offering a variety of public, private, and community services	Healthier lifestyle behaviors (e.g., diet, physical activity, sleep)
5	Brandstetter & Liebman [38]	Fidgebot	4	Nao robot	Encouraging the use of standing desks and “micro-exercises”
6	Brombacher et al. [39]	Stimulight	61	Tangible, ambient design to visualize physical activity level and share it with co-workers	Improving physical activity level of office workers
7	Cambo et al. [40]	Breaksense	6	Smartphone application using a Bluetooth beacon infrastructure and a smartwatch	Encouraging mobility during breaks
8	Damen et al. [41]	Workwalk	-	Physical outdoor walking route, which could be booked through the room booking system	Encouraging and supporting the practice of walking meetings
9	Esakia et al. [42]	FitAware	7	Three-component system including a smartwatch interface, companion application, and website.	Encouraging group cohesion in physical activity intervention
10	Fortmann et al. [43]	MoveLamp	10	Ambient light display	Moving more frequently and taking more steps each day
11	Foster et al. [44]	StepMatron	10	Facebook application, designed to provide social and competitive context for daily pedometer	Motivating physical activity in the working environment
12	Garcia et al. [45]	ESTHER 1.3	14	Android pedometer application	Active mini cycles of self-reflection on physical activity
13	Goldberg et al. [46]	Healthy Team Healthy U	466	Digital platform	Team-based health promotion and wellness program
14	Gomes et al. [47]	Steptacular	2980	Online interactive incentive system	Encouraging people to walk more
15	Grundgeiger et al. [48]	N.S.	5	Smartphone application	Combating sedentary behavior based on human movement research and distributed prospective memory
16	Güldenpfennig et al. [49]	N.S.	2	TV visualization including ambient light system	Establishing connectedness through the shared experience of positive behavior change
17	Haque et al. [50]	iGo	26	Smartphone application	Assisting employees in promoting their physical activities
18	Harjuniemi et al. [51]	Idle Stripes Shirt	-	Aesthetic, clothing-integrated display	Creating awareness of immobility periods during typical sitting-intensive office work
19	He & Agu [52]	On11	7	Smartphone application	Making people more aware of their unhealthy behaviors by highlighting sedentary behaviors
20	Hirano et al. [53]	Walkminder	8	Smartphone application using vibrations	Interrupting extended periods of inactivity and encouraging a more active lifestyle
21	Kanaoka & Mutlu [54]	N.S.	24	Humanlike NAO robot acting as a motivational agent through motivational interviewing	Increasing motivation for behavior change by talking about and reflecting on the causes of lack of motivation
22	Khot et al. (2013) [55]	Sweatatoms	-	3D-printed objects using the heartbeat pattern	Making the experience of participating in physical activity more engaging beyond screen-based feedback
23	Khot et al. (2015) [56]	EdiPulse	-	3D-printed chocolates displaying cheerful messages	Opening new interaction possibilities supporting the physical activity experience
24	Khot et al. (2015) [57]	TastyBeats	NS	A personalized sports drink representing the user’s heart rate data	Expanding the understanding technology potential to support the energy cycle when being physically active
25	Komninos et al. [58]	BeatClearWalker	20	Smartphone application including a music player	Helping users to learn how to walk at a moderate cadence
26	Lin et al. [59]	Motivate	6	Smartphone application	Providing personalized and contextualized advice on physical activities
27	Luo et al. [60]	Time for Break	25	A break-prompting system	Enabling people to set their desired work duration and prompting them to stand up or move
28	Madeira et al. [61]	Breakout	10	Ambient feedback prototype, tangible design	Recommending breaks of sedentary behavior at appropriate times
29	Mateevitsi et al. [62]	Healthbar	8	Ambient persuasive device (light)	Helping users break up their prolonged sitting habits
30	Maxhelaku et al. [63]	N.S.	-	Smartphone application	Giving information on how active people are and how they can improve their life and implement a program with activity tracker
31	Min et al. [64]	Pretty Pelvis	-	A virtual pet application	Persuading social actors for prolonged engagement toward the breaking of sedentary behavior
32	Mohadis & Ali [65]	WargaFit	8	Smartphone application	Encouraging simple physical activity, doable in an office-based environment (such as walking and stretching)
33	Moradi and Wiberg [66]	(NEAT)-Lamp and Talking Tree	6	Sensor-based lamp connected to a computer	Increasing daily movement
34	Mukhtar & Belaïd [67]	Sedentaware	4	Smartphone application	Motivating users to take corrective actions, after detecting prolonged sedentary behavior
35	Munson & Consolvo [68]	Goalpost & Goalline	23	Smartphone application	Supporting weekly physical activity goal setting and tracking.
36	Probst et al. [69]	3D Active chair	-	Interactive office chair	Reducing sedentariness through smooth integration of light physical activity into the daily work routine
37	Reeder et al. [70]	Breakbot	NS	Emotionally expressive companion robot	Encouraging employees to take breaks and socialize regularly
38	Ren et al. (2019) [71]	LightSit	50	Sensor mat embedded into an office chair and a lighting display	Helping people reduce physical inactivity and managing chronic stress at work
39	Ren et al. (2018) [72]	PCFT intervention	20	Activity tracker and smartphone application	Fitness breaks
40	Ren et al. 2019 [73]	Step-by-Step	5 + 3	A social exergame mediated by a connected cubic desk widget in a gift format	Relaying an object from a co-worker to another as a fitness task
41	Simons et al. [74]	Active Coach	130	Smartphone application	Promoting an active lifestyle
42	van Dantzig et al. [75]	Sitcoach	86	Smartphone application	Providing timely persuasive messages suggesting active breaks
43	Wai Shan Ng and Ehud Sharlin [76]	Mootchi	2	Projected avatar acting as a persuasive physical trainer	Providing users with an emotional incentive for exercising
44	Wang, Reiterer [77]	SedentaryBar	8	Context-aware reminding system using an always-on progress bar (light)	Helping screen-based workers to reduce sedentary behavior
45	Xu et al. [12]	N.S.	7	Cushion seat pan and backrest surface	Providing posture information

**Table 2:** Behavior change techniques used in the studies included in the analysis.

Behavior Change Technique	Studies
Rewards	[36,40,42,44,47,49,50,55,57,63,68,70,78]
Creating awareness/self-reflection	[36,39,43,45,51,66,67]
Social support/sharing	[35,39,41,42,46,49,68,72,73]
Goal setting	[37,40,46,52,68,72,74]
Persuasion	[34,38,62,67,79]
Education/instruction/providing information	[12,35,36,37,74]
Prompting	[45,60,80]
Self-reflection/self-monitoring	[68,74]
Tailored feedback	[36,71]
Restructuring the physical environment	[34,41]
Restructuring the social environment	[41]
Reframing beliefs	[38,41]
Gamification	[40]
Motivational interviewing	[54]
Positive feedback	[76]
Habit formation	[41]
Social cues for motivation	[38]
Competition	[44]
Not specified	[48,53,58,59,61,65,69,75]

**Table 3:** Studies defining sedentary behavior (SB) or physical activity (PA) in included interventions.

Targeted Behavior	Studies
Physical activity	[35,43,46,53,58,65,66,74,77]
Sedentary behavior	[36,45,48,60]
Not defined	[12,34,37,38,39,40,41,42,44,47,49,50,51,52,54,55,56,57,59,61,62,63,64,67,68,69,70,71,72,73,75,76]

**Table 4:** Approaches in targeted behavior in the included interventions.

Approaches I Targeted Behavior	Studies
Learning new behavior	[69]
Creating opportunities	[34,38,41,52,58,69,76]
Creating awareness	[12,35,36,37,39,40,42,43,44,45,46,47,48,49,50,51,53,54,55,56,57,59,60,62,63,64,65,66,67,68,70,71,72,73,74,75,76,77]

**Table 5:** Technology types of included interventions.

Technology Type	Studies
Lamp	[39,43,49,62,66,77]
Chair	[12,69,71,72]
Robots	[38,54,70]
3D printing	[55,56]
Phone application	[34,36,40,42,45,48,50,52,53,58,59,62,64,65,67,68,74,75]
Other, with physical component	[41,51,57,61,73]
Other, without physical component	[35,37,44,46,47,60,76]

**Table 6:** Input measures of included interventions.

Input Measures	Studies
Step count	[1,4,5,13,16,29,31,32,37,41,46,48,52,54,57,62,68,71,72,74,81]
Heartrate	[38,40,55,57,71,78]
Motion or gestures	[48,52,61,62,66,69,76,77]
Caloric intake or energy expenditure	[37,52,63]

**Figure A1:**
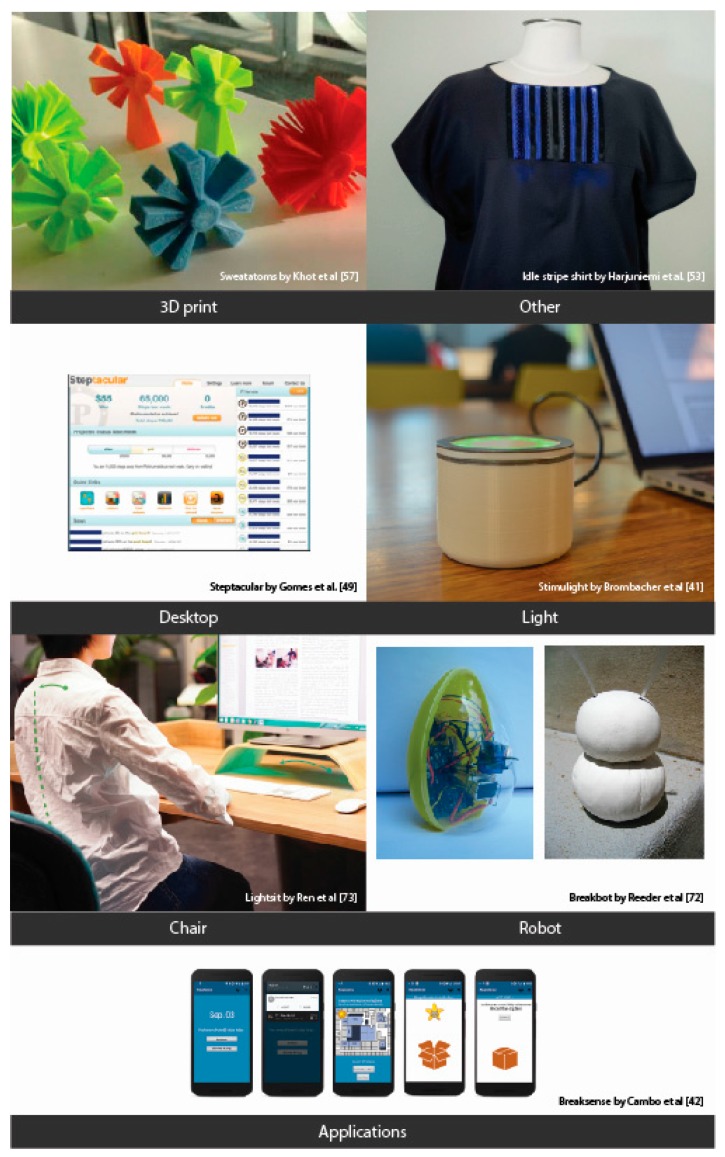
Examples of interventions categorized by technology types.
